# Distinct Associations between Hypertension and Obstructive Sleep Apnea in Male and Female Patients

**DOI:** 10.1371/journal.pone.0113076

**Published:** 2014-11-17

**Authors:** Qiang Yu, Guizhi Yin, Peng Zhang, Zhiping Song, Yueguang Chen, Dadong Zhang, Wei Hu

**Affiliations:** Department of Cardiology, the Center Hospital of Minhang District, Shanghai, China; National Institute for Viral Disease Control and Prevention, CDC, China, China

## Abstract

Obstructive sleep apnea (OSA) is highly associated with hypertension. However, the correlation between hypertension and OSA at different levels of severity and the influence of gender on that correlation are unclear. A total of 996 patients (776 males and 190 females) with OSA were recruited. The influence of gender on the correlation between hypertension and OSA at different stratifications of severity, based on the apnea-hypopnea index (AHI), was fully evaluated together with the major health risk factors obesity, age, and diabetes. Females with OSA were significantly older on average than males with OSA. Moreover, females had milder degrees of OSA on average than the extent of severity seen in males. The proportion of females with diabetes or hypertension was higher than that of males. The proportion of males with hypertension and obesity increased significantly with OSA, and age also increased with OSA. The percentage of females with hypertension at different degrees of OSA severity was stable at about 26% in the mild, moderate, and severe OSA groups. Among females, age was increased significantly in the moderate relative to the mild OSA group. Moreover, the proportion of obese subjects was increased significantly in the severe compared with the moderate OSA group. The proportions of males and females with diabetes were not significantly different among all OSA severity groups. An ordinal multivariate logistic regression analysis confirmed that hypertension, age, and obesity were associated with OSA severity in males, whereas only age and obesity were associated with OSA severity in females. Although the proportion of subjects with hypertension was higher in females with OSA than in males with OSA, the proportion of subjects with hypertension increased as the severity of OSA increased in males but not in females.

## Introduction

Obstructive sleep apnea (OSA) is a common respiratory sleep-related disease that involves the cessation or a significant decrease in airflow with breathing effort [1]. This type of sleep-disordered breathing is characterized by recurrent episodes of upper airway collapse during sleep and is associated with recurrent oxyhemoglobin desaturation and arousal from sleep [2], [3]. The prevalence of OSA is increasing both in adults and children [4], [5]. The Wisconsin Cohort Study indicated that the prevalence of OSA in subjects aged 30–60 years is 9–24% for men and 4–9% for women [5]. A 2006 study suggested that approximately 6% of adolescents have weekly sleep-disordered breathing, which is mainly OSA [6].

Normal sleep is a basic physiological process to maintain health by decreasing the body's metabolic rate, sympathetic nervous activity, blood pressure, and heart rate and increasing cardiac vagal tone [7]. OSA is now recognized not only as sleep-disordered breathing but also as a syndrome involving multiple organs that increases the risks of heart failure [8], [9], stroke [10], and cardiovascular-related mortality; moreover, it is causally related to hypertension [11]–[13].

Hypertension is also a common chronic disease that increases the risks of heart attack, stroke, kidney failure, coronary heart disease, and other serious health problems [14]. Thus the combination of OSA and hypertension can aggravate injury to the patient [15]. Observational studies show that OSA is an independent risk factor for systemic hypertension, beyond the effects of obesity, sex, and age [16]. Intermittent sustained or cyclic hypoxia, sleep-related obstructive apnea, and arousal-treated animal models have shown the causality between OSA and hypertension [17], [18]. Effective continuous positive airway pressure (CPAP) treatment in patients with moderate to severe OSA leads to a substantial reduction in day and night arterial blood pressure in patients with hypertension [11], [15]. Thus, the causal relationship between OSA and hypertension is clear.

Current clinical studies have focused mainly on the prevalence of hypertension in OSA patients [11]. Three large cross-sectional studies were reported at the end of the last century. The Wisconsin Sleep Cohort study showed that sleep-disordered breathing was associated with an increased prevalence of hypertension in employed middle-aged adults [19]. Another cross-sectional study, reported from the sleep clinic of St. Michael's Hospital in Canada, showed that an increase in the apnea-hypopnea index (AHI) by one event/h was associated with a 1% risk of having hypertension [20]. The National Institutes of Health funded Sleep Heart Health Study reported an odds ratio of 1.37 of developing hypertension using a multivariable analysis comparing the highest category of AHI ≥30/h with the lowest category (<1.5/h) [16]. In these studies, hypertension was determined to be a risk factor associated with OSA [16], [19]. However, there have been very few studies that have examined the distinct prevalent characteristics of hypertension in OSA patients according to gender. A handful of studies have reported the effect of gender on the risk of hypertension in OSA, however the results have been inconsistent [21]. One study showed that female gender is associated with hypertension among OSA patients with similar disease severity [22]. Furthermore, in that study OSA was associated with an increased prevalence of hypertension in both males and females, but the risk was higher in obese males compared with obese females with OSA [23]. Other studies showed no effect of gender on the association between hypertension and OSA [16], [24]–[26].

To determine if the prevalence of hypertension in OSA patients is influenced by gender and how gender may alter the correlation between hypertension and OSA at different levels of severity, 996 OSA patients were recruited to evaluate the association between OSA severity and hypertension, together with major health risk factors such as obesity, age, and diabetes.

## Materials and Methods

### Patients

A total of 1,113 patients with OSA (890 males and 223 females) who visited our Department of Cardiology were recruited sequentially from July 2009 to December 2013. Patients who met one of the following criteria were excluded: treatment with antihypertensive or antidiabetic drugs 1 week prior to data acquisition; treatment with CPAP or other substitution therapies; the presence of sleep disorders other than OSA; and chronic kidney disease or the use of hormone treatment. Ultimately, 966 adult patients (776 males and 190 females) were included in this study.

Written informed consent regarding the procedures and for the medical data to be used was obtained from all patients according to the guidelines of the Chinese National Ethics Regulation Committee. The review board of the Center Hospital of Minhang District approved this protocol in accordance with the amended Declaration of Helsinki (reference number: SHMHCH 2009–0009).

### Data acquisition

#### Polysomnography (PSG)

The patients arrived to our sleep lab in the early evening. Multiple channel PSG (Somnostar 4100; SensorMedics Corp., Yorba Linda, CA, USA) was conducted by full-time technicians. The technicians were responsible for attaching the electrodes and monitoring the patients. Multiple channels of data, including an electroencephalogram, electrooculogram, electromyogram, electrocardiogram, heart rhythm, pressure transducers, oxygen saturation, and chest and upper abdominal wall movement, were recorded. The apnea-hypopnea index (AHI) was calculated by dividing the number of sleep events by the number of hours of sleep. Subjects with an AHI value >5 were defined as having OSA.

#### Hypertension

Waking blood pressure was measured in the morning after overnight monitoring by PSG using a mercury sphygmomanometer. After a 5-min rest, patients in the seated position underwent three measurements at 5-min intervals, and the mean of the three measurements was calculated. Hypertension was defined as a systolic pressure >140 mmHg or diastolic pressure >90 mmHg according to American Society of Hypertension guidelines [27].

#### Diabetes

Fasting blood samples were also collected in the morning after monitoring by PSG. Serum glucose concentrations were measured with a Hitachi H-7600 Autoanalyzer (Tokyo, Japan). Diabetes was defined as a combination of a fasting plasma glucose level of ≥7.0 mmol/L, a previous diabetes diagnosis, or treatment with an anti-diabetic medication 1 week before measurement.

#### Demographics and anthropometric data

Age and sex data were collected before PSG. Anthropometric data were measured in light clothing and bare feet. Neck circumference was measured at the level of the cricothyroid membrane while the subject was standing, waist circumference was measured midway between the lower costal margin and iliac crest, and hip circumference was measured as the maximum girth at the greater trochanters. The mean of two measurements was used to calculate the subject's body mass index (BMI) and waist circumference/hip circumference ratio. Patients with a BMI >30 kg/m^2^ or waist circumference/hip circumference ratio >0.90 for males and >0.86 for females were defined as obese.

### Statistical analysis

Descriptive statistics were performed first; categorical variables are presented as frequencies (%), and non-normally distributed variables are summarized as medians and interquartile ranges. Differences in the baseline characteristics among groups were examined by the Kruskal-Wallis H test, one-way analysis of variance, Fisher's exact test, or the χ^2^ test according to the characteristics of the data distribution. P-values <0.05 were considered significant.

In the multivariate analysis, an ordinal logistic regression was performed with backward selection of variables. The AHI was used as a dependent variable; raw AHI values were inputted into the ordinal logistic regression model to evaluate the correlations between OSA severity and risk factors. Independent variables of diabetes, obesity, and hypertension were included in the ordinal logistic regression model as binary variables. The independent variable age was inputted into the ordinal logistic regression model as a continuous variable. Factors significant in the ordinal logistic regression model were tested in a stepwise multivariate model as independent variables. The level of statistical significance was set to 0.05. The statistical analysis was performed using STATA 8.0 statistical software (Stata Corp., College Station, TX, USA).

## Results

### Differences between the male and female patients

Of 966 patients with OSA who met the inclusive criteria were included in the analysis, 776 were males and 190 were females (sex ratio, 4.1). The average age of the female patients (54±12 years) was significantly higher than that of the male patients (44±17 years). The severity of OSA in the male patients (median AHI value, 38.2; interquartile range, 8.1–49.8) was generally greater than that in the female patients (median AHI value, 29.3; interquartile range, 7.4–41.2). Of the male patients, 6.1% (47/776), 12.8% (99/776), and 20.5% (159/776) had diabetes, obesity, and hypertension, respectively. Of the female patients, 10.5% (20/190), 16.8% (32/190), and 26.3% (50/190) had diabetes, obesity, and hypertension, respectively. The proportion of female OSA patients with diabetes (10.5%) was significantly higher than that of male patients (6.1%) (*p* = 0.0258). The proportion of female OSA patients with hypertension (26.3%) was higher than that of male patients (20.5%) (*p* = 0.0513).

### The proportion of subjects with hypertension and obesity and age increased with OSA exacerbation in males

**Figure 1:**
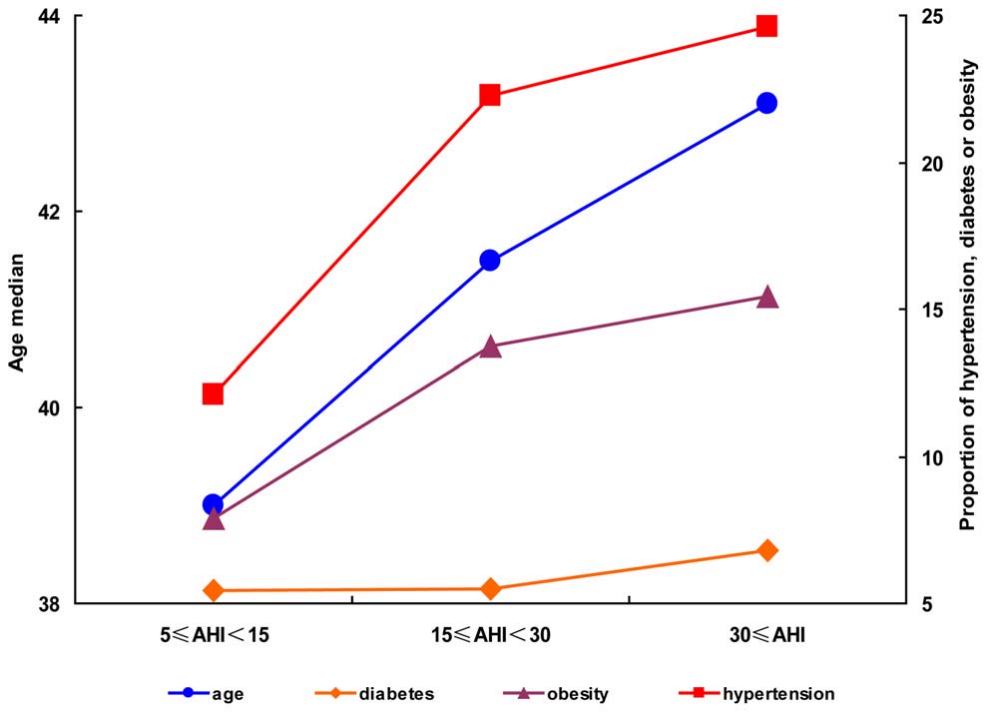
The distribution rules for age, diabetes, obesity and hypertension in male patients. The patient median ages and proportions of diabetes, obesity and hypertension were plotted against AHI severity stratifications. The mild, moderate and severe OSA groups were indicated as 5≤AHI<15, 15≤AHI<30 and AHI≥30, respectively.

The patients were stratified into mild (n = 220), moderate (n = 219), and severe (n = 337) OSA groups according to their AHI value, and the proportions with hypertension, diabetes, and obesity were plotted against the AHI stratification. The proportions of subjects with hypertension, diabetes, and obesity in the mild, moderate, and severe OSA groups were 12.3, 22.4, and 24.6%; 5.5, 5.5, and 6.8%; and 7.7, 13.7, and 15.4%, respectively (Table 1 and Figure 1). The proportion of subjects with hypertension and obesity increased significantly with aggravating OSA, whereas the proportion with diabetes was stable among the three OSA severity groups (Table 1 and Figure 1). Age also increased significantly with aggravating OSA (Table 1 and Figure 1).

**Table 1:** Baseline data from the male patients.

	Number of subjects (n = 776)	Age	Diabetes	Obesity	Hypertension	AHI
5≤AHI<15	220	39.0 (24.0∼43.0)	12 (5.5)	17 (7.7)	27 (12.3)	9.4 (5.4∼13.1)
15≤AHI<30	219	41.5 (23.0∼47.0)	12 (5.5)	30 (13.7)	49 (22.4)	21.6 (17.3∼26.2)
30≤AHI	337	43.1 (34.0∼48.0)	23 (6.8)	52 (15.4)	83 (24.6)	41.2 (35.0∼50.1)
P		<0.001	0.7334	0.0255	0.0014	<0.001

Skewed data are presented as medians (interquartile range); categorical data are presented as percentages. All ages are shown in years. Differences in baseline characteristics among the groups were examined using the Kruskal-Wallis H test, one-way ANOVA, Fisher's exact test, or χ2 test, according to the data distribution characteristics. AHI, apnea-hypopnea index.

### The proportion of subjects with hypertension did not increase with OSA exacerbation in females

**Figure 2:**
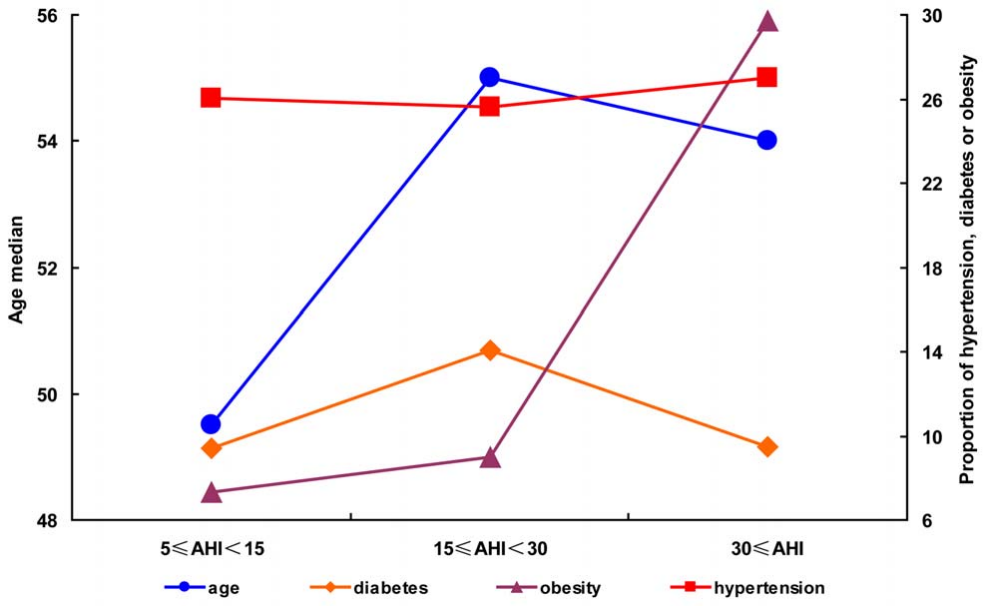
The distribution rules for age, diabetes, obesity and hypertension in female patients. The patient median ages and proportions of diabetes, obesity and hypertension were plotted against AHI severity stratifications. The mild, moderate and severe OSA groups were indicated as 5≤AHI<15, 15≤AHI<30 and AHI≥30, respectively.

The 190 female patients were also stratified into mild, moderate, and severe OSA groups according to their AHI value. There were 64, 52, and 74 patients with mild, moderate, and severe OSA, respectively. The proportions of females with hypertension and diabetes in the mild, moderate, and severe OSA groups were 26.6, 25.0, and 27.0% and 9.4, 13.5, and 9.5%, respectively (Table 2). Although the proportion of female OSA patients with (26.3%) hypertension was higher than that of male patients (20.5%), the proportion of female OSA patients with hypertension was stable at about 26% in all three OSA severity groups (Figure 2). Although the proportion of female OSA patients with diabetes increased in the moderate OSA group by 13.5% relative to the mild OSA group, no significant difference was observed among the mild, moderate and severe OSA groups (Table 2). Female age increased significantly from the mild OSA group to the moderate OSA group but decreased in the severe OSA group (Table 2 and Figure 2). The proportion of females with obesity increased from the mild OSA group to the moderate OSA group and increased significantly from the moderate OSA group to the severe OSA group (Table 2 and Figure 2).

**Table 2:** Baseline data from the female patients.

	Number of subjects (n = 190)	Age	Diabetes	Obesity	Hypertension	AHI
5≤AHI<15	64	49.5 (38.0∼56.0)	6 (9.4)	5 (7.8)	17 (26.6)	9.2 (5.8∼12.7)
15≤AHI<30	52	55.0 (46.0∼58.0)	7 (13.5)	5 (9.6)	13 (25.0)	22.1 (16.9∼27.1)
30≤AHI	74	54 (44.0∼59.0)	7 (9.5)	22 (29.7)	20 (27.0)	38.4 (32.7∼52.5)
P		<0.001	0.7189	0.0007	0.9656	<0.001

Skewed data are presented as medians (interquartile range); categorical data are presented as percentages. All ages are shown in years. Differences in baseline characteristics among the groups were examined by the Kruskal-Wallis H test, one-way ANOVA, Fisher's exact test, or χ2 test, according to the data distribution characteristics. AHI, apnea-hypopnea index.

### Hypertension was associated with OSA severity independently in males but not in females

Obesity, age, and diabetes are the main confounding factors in the correlation between OSA and hypertension. An ordinal multivariate logistic regression analysis was conducted to eliminate the confounding effects of these factors on the above proportion distributions and to reveal the correlation between hypertension and OSA severity in male and female patients. As shown in Table 3, hypertension, age, and obesity were associated with OSA severity in male patients with odds ratios (ORs) of 1.65 (95% confidence interval [CI], 1.13–3.44), 3.43 (95% CI, 2.17–5.43), and 4.10 (95% CI, 2.34–7.32), respectively, suggesting that hypertension, age, and obesity are independently associated with OSA severity. In contrast, hypertension was not associated with OSA severity in females, but age and obesity were associated with OSA severity with ORs of 1.44 (95% CI, 1.17–4.66) and 3.79 (95% CI, 1.54–8.23), respectively. Diabetes was not associated with OSA severity in male or female patients.

**Table 3:** Adjusted odds ratios (ORs) relating OSA severity to various risk factors.

	Males	Females
Age per 10-year increment	1.65 (1.13∼3.44)	1.44 (1.17∼4.66)
Hypertension	3.43 (2.17∼5.43)	/
Obesity	4.10 (2.34∼7.32)	3.79 (1.54∼8.23)

ORs (95% CIs), adjusted for each covariate for the effects of other covariates, were evaluated by ordinal logistic regression modeling.

## Discussion

Population-based studies have identified an independent correlation between a higher AHI value and increased blood pressure [16], [19], but how hypertension is correlated with OSA severity and how gender alters that correlation is still unclear. In this study, we demonstrated that 1) the overall proportion of female OSA patients with hypertension was higher than that of male OSA patients, and 2) the proportion of patients with OSA and hypertension increased significantly as the degree of OSA worsened in male patients, whereas it was stable in female patients. These results were further confirmed in the ordinal logistic regression model after adjusting for each covariate. Our data revealed that OSA is associated with hypertension, with distinct mechanisms in male and female OSA patients. This emphasized the existence of gender-specific discrepancies in the pathophysiologic process of OSA relative to hypertension. As we described in the previous section, the effect of gender on the risk of hypertension in OSA patients has been reportedly inconsistent [21], and this inconsistency might be due to small sample size, differences in the study population composition and a non-targeted research design [28]. Our data does not conflict entirely with previous reports. In the study by Sforza et al., females were found to have an increased risk of hypertension (OR, 1.52) compared with males; however, this study simply compared hypertension events between females and males and did not examine the correlation between the proportion of subjects with hypertension and OSA severity according to gender [29].

The key challenge in deciphering OSA-associated risk factors, including hypertension, is appropriately accounting for the many confounding variables, particularly obesity and age [28], [30]. This situation is due to the complex correlations among these parameters, but study design is also critical to reduce interference caused by confounding factors. Although no current statistical approach can satisfactorily address the complex interaction among risk factors correlated with a certain disease, applying the appropriate statistical analysis might reveal the key question. For this reason, we selected the main health factors of age, diabetes, obesity and hypertension to investigate the correlation between OSA and hypertension. We believe any confounding effect might be reflected by these major factors and can be adjusted using the ordinal multivariate logistic regression analysis. In this study, we concentrated on patients with OSA, and its correlation with risk factors was revealed by stratifying for both OSA severity and gender. Our results provide an interesting research direction for OSA relative to hypertension, which is why the proportion of female patients with OSA and hypertension was higher than that of males and why the proportion of hypertension increased with OSA severity only in males. In addition to hypertension, age and obesity were associated with OSA severity independently in males. Among the female patients, only age and obesity were independently associated with OSA severity. Although the proportion of female patients with OSA and diabetes was significantly higher than that of male patients with OSA, diabetes was not associated with OSA severity in males or females.

Reports indicate that as many as 50% of patients with hypertension have concomitant OSA [31]. Although we did not calculate the ratio in this way, our data show that 21.0% (26.3% of females and 20.5% of males) of the patients with OSA had concurrent hypertension. Many studies have demonstrated that OSA induces hypertension [19]–[23], [32]. The decrease in nocturnal blood pressure that occurs in normal individuals is altered in patients with OSA [33]. Isolated systolic hypertension, which is more commonly seen in elderly patients, is not associated with OSA in any age group [25]. These confusing conclusions are still controversial [28]. In our study, all patients suspected to have hypertension were confirmed in the morning after over-night monitoring by PSG. The diagnosis of hypertension was based on individual history and nosocomial confirmation, and patients were diagnosed with hypertension regardless of their systolic or diastolic pressure. This definition might conceal the detailed effects of OSA severity on systolic or diastolic pressure. We plan to perform 24-hour ambulatory blood pressure monitoring on patients with OSA to assess the effects of OSA on nighttime and daytime blood pressure readings.

Sex affects the incidence of the disease, OSA being more prevalent in males than in females [29], [34]. The reason for the distinct associations between hypertension and OSA in male and female patients is hard to explain currently, the body fat distribution characteristic and hormonal influences might be the reasons for the distinct correlations between gender and OSA and relative complications. Menopause is a risk factor for OSA [34], [35]. In our study, major of the females patients are at menopause period, the medians and interquartile range are 54 and 39 to 59, so the impact of menopause on the correlation between OSA and hypertension need further evaluation.

## Conclusions

The proportion of subjects with hypertension was higher in females than males with OSA The proportion of subjects with hypertension increased as the severity of OSA increased in males but not in females.
